# Two Cases of Delayed Diagnosis of Leprosy in Mauritania

**DOI:** 10.1155/2018/4394297

**Published:** 2018-05-16

**Authors:** Boushab Mohamed Boushab, Fatima-Zahra Fall-Malick, Leonardo K. Basco

**Affiliations:** ^1^Department of Internal Medicine and Infectious Diseases, Kiffa Regional Hospital, Assaba, Mauritania; ^2^National Institute of Hepatitis and Virology, School of Medicine, Nouakchott, Mauritania; ^3^Aix Marseille Univ, IRD, AP-HM, SSA, VITROME, IHU-Méditerranée Infection, Marseille, France

## Abstract

Leprosy is a chronic infectious disease that mainly affects the skin, mucous membranes, and peripheral nervous system. The clinical manifestations of leprosy are numerous and polymorphic with the most frequent signs involving skin and neurological damage. Some of its manifestations, such as joint pain, are unusual. Its elimination as a public health problem in many countries seems to lead to a lack of practical knowledge among health care personnel and as a consequence a risk of late diagnosis. As in other countries, leprosy has become rare in Mauritania. We report two cases of misdiagnosed leprosy in two male patients aged 17 and 65 years. Clinical manifestations included polyarthritis, bilateral plantar perforation, and severely deformed hands and feet in the first case and lichenoid lesions, hypopigmented papules, and unilateral bronchial rales in the second case. The duration of development and persistence of clinical signs before establishment of correct diagnosis was seven to ten years despite the presence of anesthetic, hypochromic maculopapular skin lesions and neurologic signs suggestive of leprosy in both cases. A multilevel chemotherapeutic regimen recommended by the World Health Organization (WHO) was effective, and the patients' condition evolved satisfactorily. The scarcity of leprosy in our health care facilities often leads to a wrong diagnosis. It is imperative to inform physicians to increase their vigilance for appropriate screening and reporting of these cases. The prognosis depends largely on early diagnosis and appropriate treatment.

## 1. Introduction

Leprosy, or Hansen's disease, is a chronic infectious disease caused by the bacillus* Mycobacterium leprae*, which mainly affects the skin, mucous membranes, and peripheral nervous system [1]. Transmitted by droplets of buccal or nasal origin during close and frequent contact with an infected and untreated subject [2], this disease may manifest itself as a result of motor sequelae in relation to neurological or mutilating bone damage and visceral dissemination that can be life threatening [3]. Although leprosy still occurs with a relatively high prevalence in some countries in Asia and South America [1], leprosy has become a rare disease in Mauritania. We report here two cases of late diagnosis of leprosy despite the persistence of pathognomonic signs.

## 2. Case Report 1

A 17-year-old farmer residing in Kankossa (Kankossa/Assaba region), Mauritania, spontaneously presented for consultation at Kiffa Regional Hospital on December 2, 2016, for severe chronic arthralgia affecting joints of the hands associated with bilateral perforating plantar ulcer. The patient's clinical history showed that the cutaneous and neurological signs had evolved for 7 years, which initially presented with hypopigmented maculopapular lesions and paresthesia at the interphalangeal extremities and on the plantar surfaces of the feet. Prior to consultation in our hospital, the patient had seen several dermatologists and traditional healers over the past 7 years. None of these practitioners considered leprosy for differential diagnosis, and the prescribed analgesic, antibiotic, and topical treatments had not improved the patient's condition.

**Figure 1:**
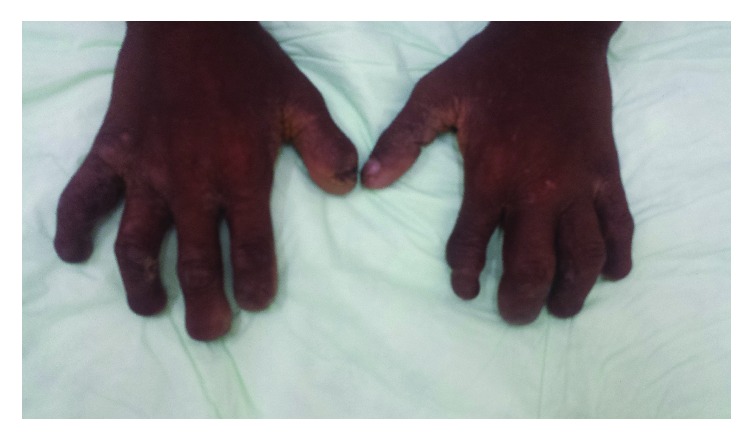
Bilateral atrophied hands with hypopigmented and hypoesthesic lesions in interdigital spaces.

**Figure 2:**
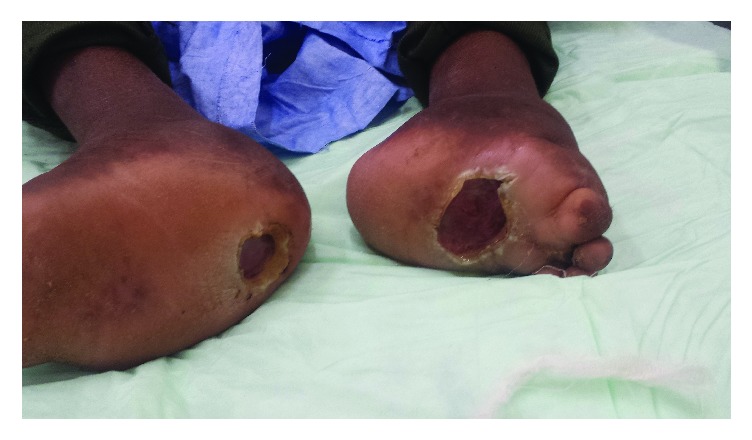
Bilateral neuropathic plantar ulcer.

Physical examination revealed anesthetic distal phalangeal atrophy associated with severe deformity of the hands, hypochromic, anesthetic macular lesions (Figure 1), atrophy of the metatarsals and phalanges with severe deformity of the feet, and bilateral plantar perforations (Figure 2). Hypertrophied nerve trunks were palpable at the ulnar and popliteal level. Visual examination was normal. Clinical diagnosis of lepromatous leprosy was established on the basis of 2 of 3 criteria set by the World Health Organization (WHO) [1]: hypochromic anesthetic skin lesions and palpable peripheral nerves. The third criterion, bacteriological examination of skin smears and/or skin biopsy of infiltrated lesions, was not sought to demonstrate the presence of* M. leprae* since these examinations are not available in the country.

**Figure 3:**
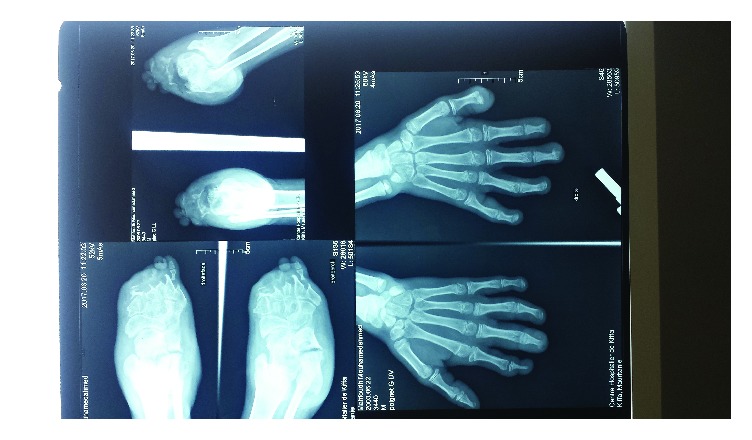
Bilateral osteoporosis.

Human immunodeficiency virus (HIV) serology was negative. The radiographs of the hands and feet showed osteoporosis (Figure 3). After routine pretherapeutic laboratory examinations that proved to be normal, antileprosy treatment (rifampicin 600 mg/day, dapsone 100 mg/day, and clofazimine 100 mg/day) was prescribed and was generally well tolerated, resulting in good clinical progress. After six months of therapy, the superficial skin lesions regressed and were completely healed. No relapse was noted during the follow-up period.

## 3. Case Report 2

**Figure 4:**
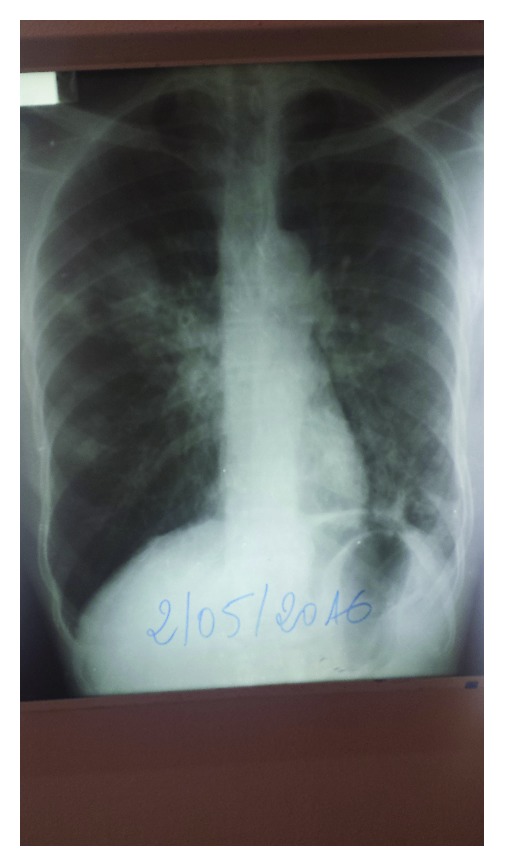
Reticulonodular infiltrates at the apex of the right lungs associated with bilateral hilar lymphadenopathy.

A 65-year-old farmer residing in Gougui (Kobeni/Hodh El Gharbi region), Mauritania, consulted on December 2, 2016, at the Aïoun Regional Hospital for maculopapular, nodular lesions associated with chest pain and chronic productive cough. On admission to hospital, the skin signs had evolved over the past ten years, at first consisting of lichenoid lesions and multiple hypopigmented papules. A few months before consultation, a productive cough associated with progressive deterioration of the general condition occurred. There was no history of tuberculosis. Various analgesics, antibiotics, and topical treatment, including traditional medicines, had not improved his condition. The physical examination revealed the presence of diffuse hypopigmented lichenoid lesions varying from 2 to 5 cm in diameter at the base of the nose with sensory loss. Visual examination was normal. Auscultation revealed rales over the right bronchus. Sputum examination and Ziehl-Neelsen skin smears were negative. Skin biopsy was not performed due to inadequate medical facilities. HIV serology was negative. Laboratory examinations showed anemia (9 g/dl hemoglobin), normal blood sugar, and normal renal and hepatic functions. The chest X-ray showed reticulonodular infiltrates at the right lung apex and bilateral hilar lymphadenopathy (Figure 4). The diagnosis of lepromatous leprosy with concomitant disseminated tuberculosis was made. Antileprosy and antituberculosis treatment (rifampicin 600 mg/day, dapsone 100 mg/day, and clofazimine 100 mg/day) was initiated and was generally well tolerated, leading to a favorable response.

## 4. Discussion

In the 1990s, WHO set the goal of eliminating leprosy as a public health problem from 2000 to 2005, with a prevalence of less than 1 for 10,000 inhabitants. WHO also recommended the establishment of a leprosy surveillance system in endemic countries in order to have indicators for the detection, management, and follow-up of patients [4]. The successful programme for control and elimination of this disease as a public health problem in some countries, such as Mauritania, seems to generate ignorance of leprosy among health personnel and, as a consequence, may increase the risk of late diagnosis, as in our cases.

The peculiarities of our observations were the long duration of disease evolution before correct clinical diagnosis due to initial misdiagnosis, and in our first case, severe deformity of hands and feet, including bilateral deeply perforating plantar ulcer. Delays in the duration of diagnosis were also noted in Senegal and France [3, 5, 6]. Indeed, articular pain, atrophy of the fingers, and polymorphic cutaneous manifestations often evoke rheumatoid arthritis or deforming polyarthritis. However, the diagnosis of musculoskeletal injury due to leprosy is difficult, given its multiple manifestations and the fact that many autoimmune disorders are part of differential diagnosis [7]. The confusion between leprosy and rheumatic disease has already been reported in the literature [8]. The appearance of “pudgy” deformed fingers is certainly misleading, but it may constitute an unsuspected inaugural form of leprosy, especially in its lepromatous form [9]. The perforating plantar ulcer in our first patient initially evoked a diabetic foot, but its bilateral character and hypertrophy of the ulnar and popliteal nerve trunks helped orient our diagnosis toward leprosy. Plantar neuropathic ulcer is a common and frequent complication of diabetes, a common pathology recognized as a pandemic by the WHO. Although frequently encountered during medical practice, the pathophysiology of neuropathic ulcer associated with diabetes remains poorly understood [10]. In our first patient, the presence of plantar perforation is an indirect evidence for the long duration of evolution but is also a risk of tetanus, osteitis, and sepsis. The hypochromic macules alone were not suggestive of leprosy, but their anesthetic character associated with nerve hypertrophy indicated leprosy in our first case.

In the second case, a diagnosis of pulmonary tuberculosis was suggested by the presence of persistent cough and radiological signs despite a well-conducted antibiotic therapy. As for the diagnosis of leprosy, lichenoid lesions and diffuse hypopigmented papules alone were not highly suggestive of leprosy but their anesthetic character was decisive to establish the correct diagnosis.

Leprosy and tuberculosis coinfection is very rare. However, the earliest case of coinfection of leprosy and tuberculosis was detected in the deoxyribonucleic acid of a man discovered in a burial site dating from the first century [8]. In our second case, leprosy had actually preceded tuberculosis, in agreement with earlier reports of several authors [8, 11, 12]. To our knowledge, there has been no reported case of concomitant tuberculosis and leprosy in Mauritania. The duration between the development of leprosy and tuberculosis varied from 2 months to 15 years [12]. In our case, it was 10 years. However, in one study, it was reported that two cases of tuberculosis occurred before leprosy, and it was suggested that tuberculosis can occur together with the full spectrum of leprosy [9]. The interaction between leprosy and tuberculosis and their impact on the incidence of the other remain a subject of debate [10, 13]. Some authors have observed that cases of pulmonary tuberculosis developed after taking a corticosteroid used primarily for the treatment of silent neuropathy associated with leprosy [11, 13].

Misdiagnosis was due to the rarity of leprosy cases seen in health care facilities in Mauritania today and the “atypical” nature of the lesions in the two cases reported here. Leprosy may become a global health problem due to the reactivation of latent, previously undiagnosed cases, even in the Western world, due to the use of strong immunosuppressive regimens for various diseases [14]. In cases of doubt, a skin smear and/or biopsy often helps to establish the final diagnosis [1]. At the country level, central laboratory with the capacity to perform microbiological diagnosis of tuberculosis and leprosy is still required. In our cases, skin biopsy was not done due to inadequate health resources, but the clinical manifestations were typical and obvious, fulfilling 2 of 3 criteria established by WHO. Treatment consisted of multidrug therapy as recommended by WHO, leading to favorable outcomes in our patients.

## 5. Conclusion

The polymorphic character of leprosy and the lack of knowledge about its clinical manifestations among most health care personnel due to the reduction of its prevalence constitute a source of misdiagnosis. Our reported cases illustrate these points. Early detection as recommended by WHO should reduce the occurrence of new active cases in the community. To attain the goal of elimination of leprosy, it is imperative for physicians and nurses to increase their vigilance in screening, treating, and reporting cases.

## References

[1] Eichelmann K., González González S. E., Salas-Alanis J. C., Ocampo-Candiani J. (2013). Leprosy. An update: Definition, pathogenesis, classification, diagnosis, and treatment.

[2] (2012). Global leprosy situation.

[3] Niang S. O., Diallo M., Ndiaye M. (2011). Epidemiologic and clinicopathologic aspects of Leprosy in Dakar; evaluation of 73 new cases.

[4] World Health Organization (2009). Global leprosy situation.

[5] Donoghue H. D., Marcsik A., Matheson C. (2005). Co-infection of Mycobacterium tuberculosis and Mycobacterium leprae in human archaeological samples: A possible explanation for the historical decline of leprosy.

[6] Prasad R., Verma S. K., Singh R., Hosmane G. (2010). Concomittant pulmonary tuberculosis and borderline leprosy with type-II lepra reaction in single patient.

[7] Sendrasoa F. A., Ranaivo I. M., Raharolahy O., Andrianarison M., Ramarozatovo L. S., Rapelanoro Rabenja F. (2015). Pulmonary Tuberculosis and Lepromatous Leprosy Coinfection.

[8] Agarwal D. K., Mehta A. R., Sharma A. P. (2000). Coinfection with leprosy and tuberculosis in a renal transplant recipient.

[9] Rao G. R., Sandhya S., Sridevi M., Amareswar A., Narayana B. L., Shantisri (2011). Lupus vulgaris and borderline tuberculoid leprosy: an interesting co-occurrence.

[10] Rawson T. M. I., Anjum V., Hodgson J. (2014). Leprosy and tuberculosis concomitant infection: a poorly understood, age-old relationship.

[11] Prasad S., Misra R., Aggarwal A. (2013). Leprosy revealed in a rheumatology clinic: a case series.

[12] Meyer M., Ingen-Housz-Oro S., Ighilahriz O. (2008). Polyarthritis and papular eruption revealing leprosy.

[13] Kuntz J. L., Meyer R., Vautravers P., Kieffer D., Asch L. (1979). Polyarthritis in leprosy.

[14] Vanlerberghe B., Devemy F., Duhamel A., Guerreschi P., Torabi D. (2014). Conservative surgical treatment for diabetic foot ulcers under the metatarsal heads. A retrospective case-control study.

